# RETRACTION: Preparation and Multitarget Anti‐AD Activity Study of Chondroitin Sulfate Lithium in AD Mice Induced by Combination of D‐Gal/AlCl_3_

**DOI:** 10.1155/omcl/9763163

**Published:** 2026-03-16

**Authors:** Oxidative Medicine and Cellular Longevity

RETRACTION: D. Gao, P. Li, F. Gao, et al., “Preparation and Multitarget Anti‐AD Activity Study of Chondroitin Sulfate Lithium in AD Mice Induced by Combination of D‐Gal/AlCl_3_,” *Oxidative Medicine and Cellular Longevity*, 2022, 9466166, https://doi.org/10.1155/2022/9466166.

The above article, published online on 12 November 2022 in Wiley Online Library (wileyonlinelibrary.com), has been retracted by agreement between the journal Editor‐in‐Chief, Jeannette Vasquez Vivar; and John Wiley & Sons Ltd. The retraction has been agreed due to multiple inconsistencies identified in the figures. The authors originally requested to correct Figure 10, however subsequent investigation identified concerns with Figures 4–6, 9, and 10.

Specifically:

• The authors confirmed that the below is incorrect in Figure 10
  ◦ The graph of the TNF‐alpha bands (3rd, 4th, 5th bars)
  ◦ The graph of the p‐Erk bands
• The CA2 400x/H‐CS‐Li panel of Figure 4 contains unexpected repeated elements when compared to the CA3 400x/H‐CS‐Li panel in Figure 4.
• The CA4 100x/Control panel of Figure 4 contains unexpected repeated elements when compared to the DG 100x/Control panel in Figure 4.
• The CA4 100x/LiCl panel of Figure 4 contains unexpected repeated elements when compared to the DG 100x/LiCl panel in Figure 4.
• The CA1 100x/Control panel of Figure 5 appears to contain unexpected repeated elements when compared to the CA2 100x/Control panel in Figure 5.
• The CA2 100x/Control panel of Figure 5 appears to contain unexpected repeated elements when compared to the CA3 100x/Control panel in Figure 5.
• The CA4 100x/LiCl panel of Figure 6 appears to contain unexpected repeated elements when compared to the DG 100x/Control panel in Figure 6.
• The second set of GAPDH bands in Figure 9 appear identical to the fifth set of GAPDH bands shown in Figure 10.

The author’s response was assessed by the editorial board who deemed the results now unreliable, and therefore the article is retracted.

The authors disagree with the retraction.

